# Supplementary material for: Lymphatic filariasis epidemiology in Samoa in 2018: Geographic clustering and higher antigen prevalence in older age groups
Source: PLoS Negl Trop Dis. 2020 Dec 21;14(12):e0008927. doi: 10.1371/journal.pntd.0008927 (PMC7785238; doi:10.1371/journal.pntd.0008927)
Supplement: S1 Text — (DOCX) [file pntd.0008927.s001.docx]

**SUPPLEMENTARY MATERIALS**

**S1 Text. Adjustment for sampling design**

Selection probability by household, individual and village

*Probability of household selection:* For each village, the actual number of households surveyed in the village was divided by the total number of households reported in the 2016 census. This probability was applied to all individuals aged ≥10 years.

*Probability of individual selection for children aged 5-9 years*: Estimated from the number of children aged 5-9 years actually sampled (sum from both convenience and household surveys), divided by the approximate number in that each group in each village (based on total village population and population age distribution in 2016 census). In one purposive PSU (Salua on Manono Island) and one random PSU (Sagone), the actual number of children sampled exceeded the predicted number of children in that age group (by 2.25 and 1.01 times), thus the selection probability was adjusted to 1.

*Sampling completeness of persons within households* was estimated from the number of persons tested divided by the number of household residents aged ≥5 years. In some households, the number of persons tested exceeded the number of reported residents (because those who slept there the previous night were also eligible); in which case the denominator of persons per household was revised upwards and the selection probability adjusted down to 1.

*Probability of village selection:* The five purposive villages were given a selection probability of 1; the randomly selected villages had a probability of selection of 0.114114 (38 villages selected out of the remaining 333 villages in Samoa at the 2016 census). Systematic selection was done using a complete list of villages in all regions, with a random start.

Survey design

Since this was a cluster survey (with PSUs as clusters), adjustment for survey design was accomplished by assigning PSU as the primary sampling unit in STATA svyset command.

Age and gender standardization

Age weightings by 5-year age groups (from age ≥5 years to ≥75 years) and gender (51.5% male and 48.5% female) were assigned to each individual based on 2016 census data.

Tables summarizing the adjustments and those used for each analysis are given in S2 and S3 Tables.
