# Supplementary material for: Lymphatic filariasis epidemiology in Samoa in 2018: Geographic clustering and higher antigen prevalence in older age groups
Source: PLoS Negl Trop Dis. 2020 Dec 21;14(12):e0008927. doi: 10.1371/journal.pntd.0008927 (PMC7785238; doi:10.1371/journal.pntd.0008927)
Supplement: S1 Table — (DOCX) [file pntd.0008927.s002.docx]

**S1 Table. Parameters used for adjustments and standardization**

|  | Subgroup | Probability of selection | Notes |
| --- | --- | --- | --- |
| Gender | Male  Female | 0.515  0.485 |  |
| Villages | Random  Purposive | 0.114114  1.0 |  |
| PSUs | Random  Purposive | 0.114114  1.0 |  |
| Households | By PSU | Range 0.0244698 to 0.3333333 |  |
| Individuals | 5-9 years by PSU  ≥10 years by PSU | Range 0.1087379 to 1.0  Range 0.02447698 to 0.3333333 |  |
| Sampling completeness (FTS done) within households | Persons ≥5 years | Range 0 to 1 | More than 97% of inhabitants were successfully tested in 97% of households. |
|  |  |  |  |
| Age groups (years) | Male  (proportion of total population) | Female  (proportion of total population) | Total |
| 5-9 | 0.07373 | 0.069435 | 0.122712 |
| 10-14 | 0.070444 | 0.066341 | 0.117243 |
| 15-19 | 0.063395 | 0.059702 | 0.105511 |
| 20-24 | 0.047459 | 0.044694 | 0.078987 |
| 25-29 | 0.040849 | 0.038469 | 0.067986 |
| 30-34 | 0.036932 | 0.034781 | 0.061468 |
| 35-39 | 0.034801 | 0.032774 | 0.057921 |
| 40-44 | 0.03339 | 0.031445 | 0.055573 |
| 45-49 | 0.028434 | 0.026778 | 0.047324 |
| 50-54 | 0.02424 | 0.022828 | 0.040343 |
| 55-59 | 0.01889 | 0.017789 | 0.031439 |
| 60-64 | 0.012728 | 0.011986 | 0.021183 |
| 65-69 | 0.010795 | 0.010166 | 0.017967 |
| 70-74 | 0.008181 | 0.007704 | 0.013616 |
| 75+ | 0.010731 | 0.010106 | 0.01786 |
