# Supplementary material for: Lymphatic filariasis epidemiology in Samoa in 2018: Geographic clustering and higher antigen prevalence in older age groups
Source: PLoS Negl Trop Dis. 2020 Dec 21;14(12):e0008927. doi: 10.1371/journal.pntd.0008927 (PMC7785238; doi:10.1371/journal.pntd.0008927)
Supplement: S2 Table — (DOCX) [file pntd.0008927.s003.docx]

**S2 Table. Adjustments and standardization used for different estimates**

| Ag or Mf prevalence estimates | Age | Gender | Household Selection Probability | Child Selection Probability | Clustering by PSU |
| --- | --- | --- | --- | --- | --- |
|  |  |  |  |  |  |
| Age-specific (5-9 years) | No | Yes | No | Yes | Yes |
| Age-specific (≥10 years) | No | Yes | Yes | No | Yes |
| Age and gender specific (5-9 years) | No | No | No | Yes | Yes |
| Age and gender specific (≥10 years) | No | No | Yes | No | Yes |
| All ages (≥5 years) | Yes | Yes | Yes | Yes | Yes |
| All ages (≥5 years) by region | Yes | Yes | Yes | Yes | Yes |
| All ages (≥5 years) by PSU | Yes | Yes | Yes | Yes | No |
