# Supplementary figures and images for: Lymphatic filariasis epidemiology in Samoa in 2018: Geographic clustering and higher antigen prevalence in older age groups
Source: PLoS Negl Trop Dis. 2020 Dec 21;14(12):e0008927. doi: 10.1371/journal.pntd.0008927 (PMC7785238; doi:10.1371/journal.pntd.0008927)

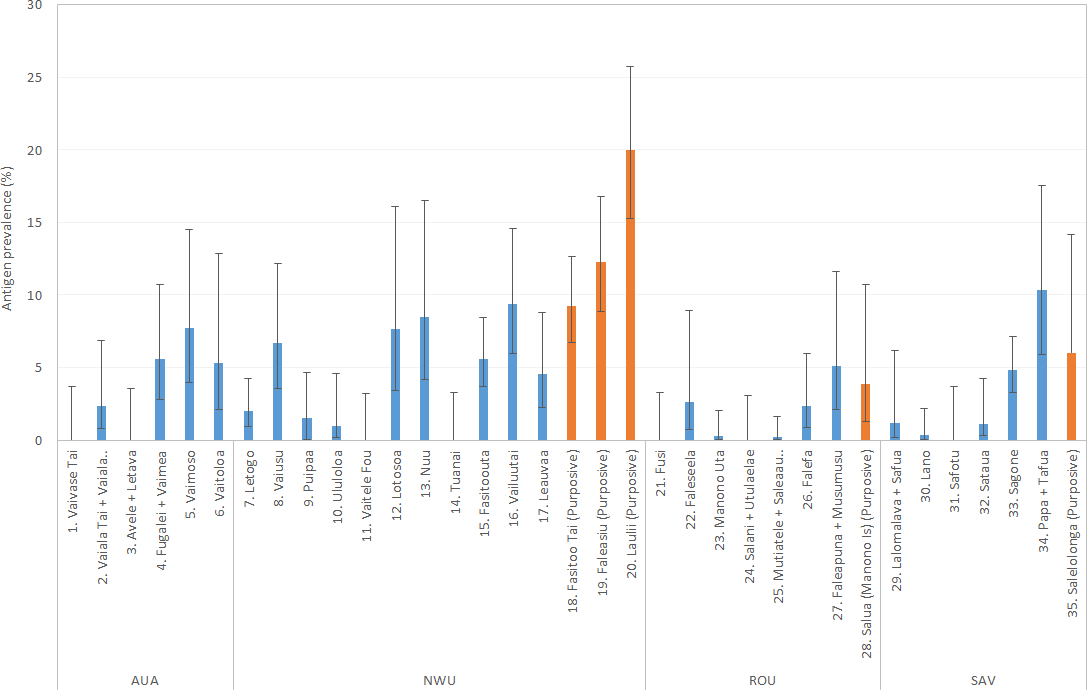

Supplement: S1 Fig — (TIF) [file pntd.0008927.s004.tif]

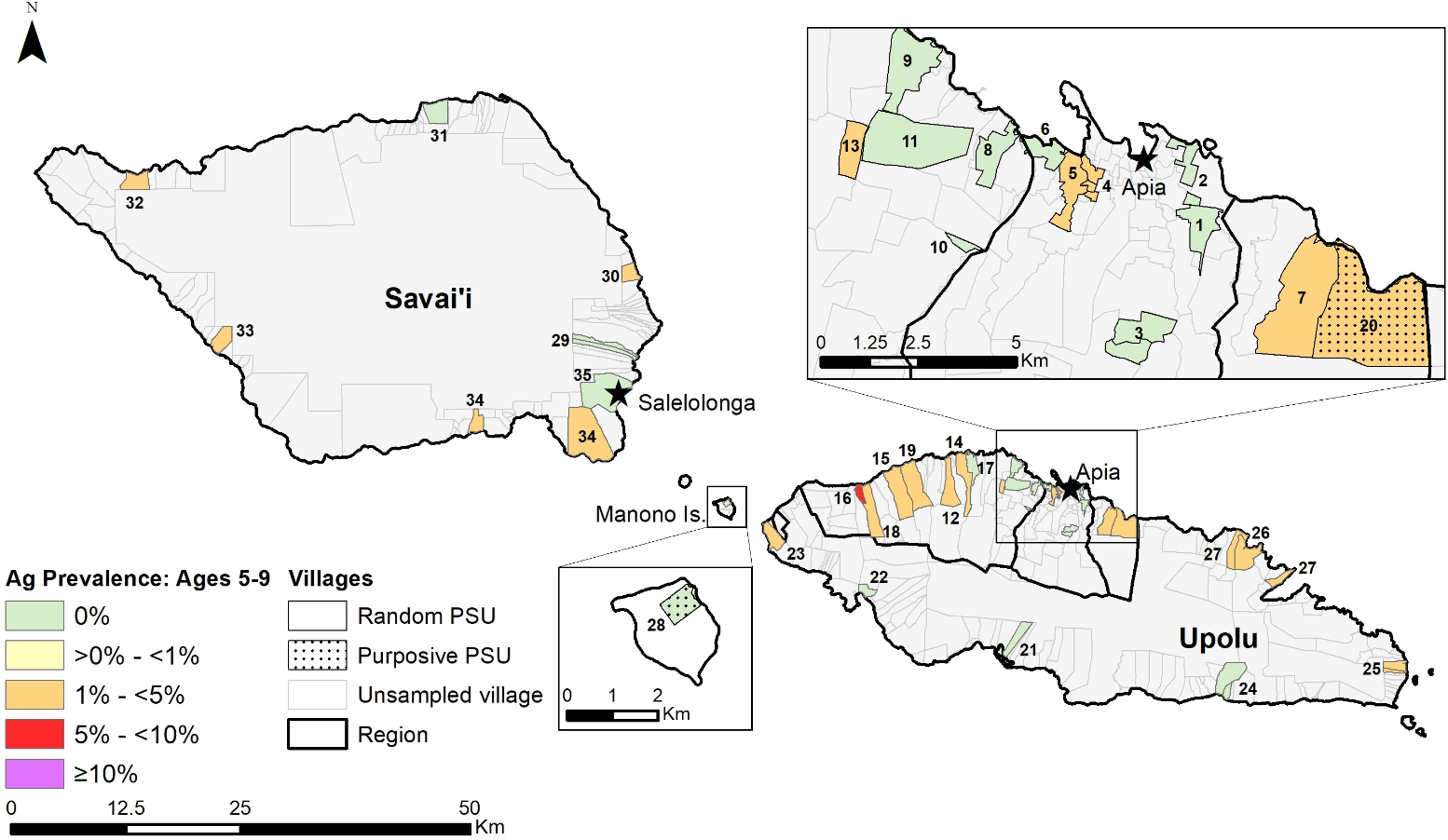

Supplement: S2 Fig — (TIF) [file pntd.0008927.s005.tif]

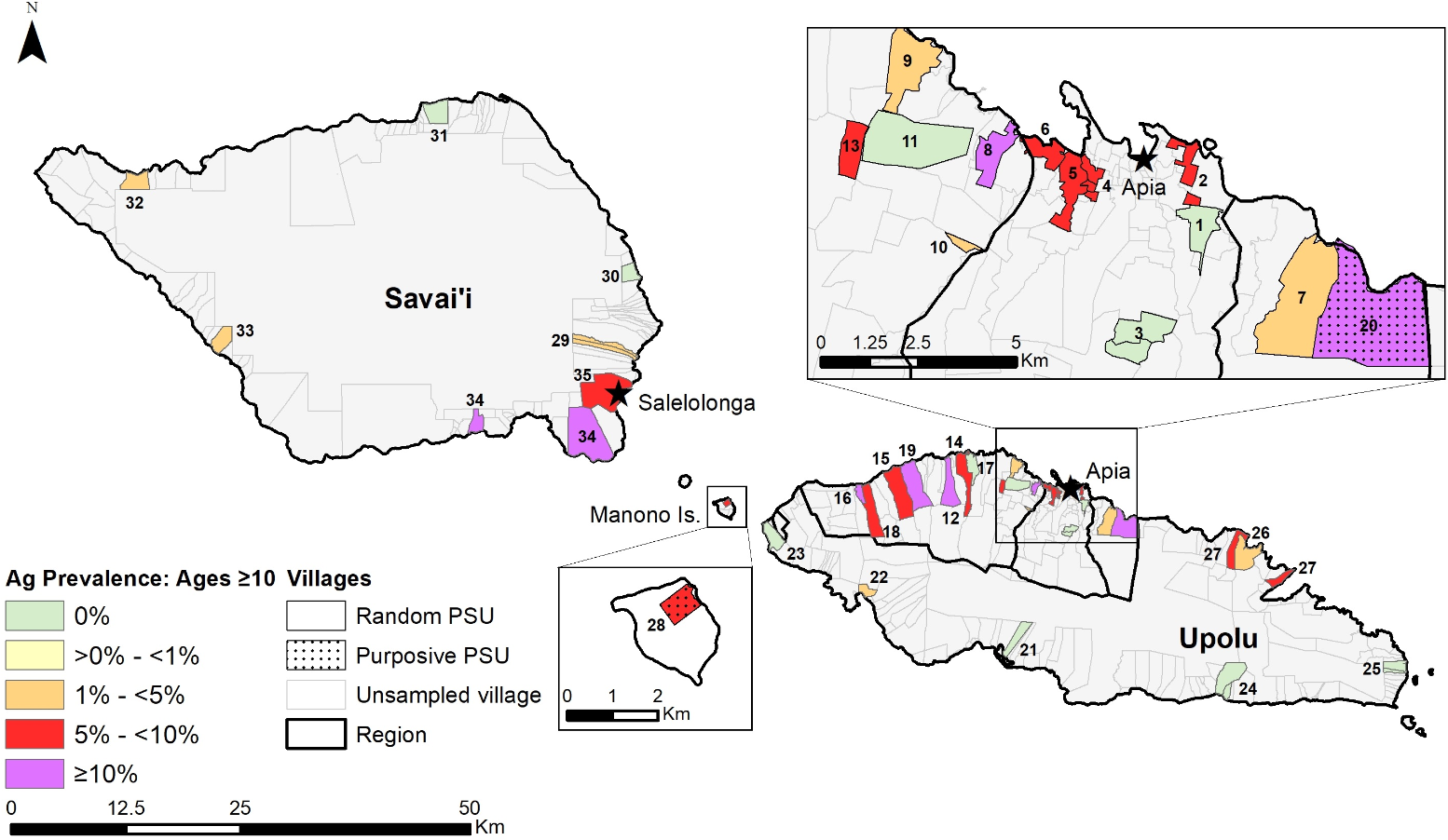

Supplement: S3 Fig — (TIF) [file pntd.0008927.s006.tif]
