# Supplementary material for: Lymphatic filariasis epidemiology in Samoa in 2018: Geographic clustering and higher antigen prevalence in older age groups
Source: PLoS Negl Trop Dis. 2020 Dec 21;14(12):e0008927. doi: 10.1371/journal.pntd.0008927 (PMC7785238; doi:10.1371/journal.pntd.0008927)
Supplement: S1 Checklist — (DOCX) [file pntd.0008927.s007.docx]

**S1 Checklist. STROBE (Strengthening the Reporting of Observational Studies in Epidemiology) Checklist for *cross-sectional studies***

| **Section and Item** | Item No | Recommendation | Page |
| --- | --- | --- | --- |
| **Title and abstract** | 1 | (*a*) Indicate the study’s design with a commonly used term in the title or the abstract | Page 2 |
|  |  | (*b*) Provide in the abstract an informative and balanced summary of what was done and what was found | Page 2 -3 |
| Introduction | | | |
| Background/rationale | 2 | Explain the scientific background and rationale for the investigation being reported | Page 4-5 |
| Objectives | 3 | State specific objectives, including any prespecified hypotheses | Page 6-7 |
| Methods | | | |
| Study design | 4 | Present key elements of study design early in the paper | Page 8 |
| Setting | 5 | Describe the setting, locations, and relevant dates, including periods of recruitment, exposure, follow-up, and data collection | Setting and location: page 7-8, Figure 1  Periods of recruitment: page 8, 13  Data collection: page 11-12 |
| Participants | 6 | (*a*) Give the eligibility criteria, and the sources and methods of selection of participants | Selecting households and children’s survey: page 10-12  Eligibility criteria: page 10 |
| Variables | 7 | Clearly define all outcomes, exposures, predictors, potential confounders, and effect modifiers. Give diagnostic criteria, if applicable | Pages 14-15 |
| Data sources/ measurement | 8 | For each variable of interest, give sources of data and details of methods of assessment (measurement). Describe comparability of assessment methods if there is more than one group | Pages 10-13 |
| Bias | 9 | Describe any efforts to address potential sources of bias | Page 11, page 14, Supp Tables S1 and S2 |
| Study size | 10 | Explain how the study size was arrived at | Page 10 |
| Quantitative variables | 11 | Explain how quantitative variables were handled in the analyses. If applicable, describe which groupings were chosen and why | Page 14-15 |
| Statistical methods | 12 | (*a*) Describe all statistical methods, including those used to control for confounding | Page 14-15 |
|  |  | (*b*) Describe any methods used to examine subgroups and interactions | Page 14 |
|  |  | (*c*) Explain how missing data were addressed | N/A |
|  |  | (*d*) If applicable, describe analytical methods taking account of sampling strategy | N/A |
|  |  | (*e*) Describe any sensitivity analyses | N/A |
| Participants | 13* | (a) Report numbers of individuals at each stage of study—eg numbers potentially eligible, examined for eligibility, confirmed eligible, included in the study, completing follow-up, and analysed | Page 15 -18 |
|  |  | (b) Give reasons for non-participation at each stage | Page 18 |
|  |  | (c) Consider use of a flow diagram | N/A |
| Descriptive data | 14* | (a) Give characteristics of study participants (eg demographic, clinical, social) and information on exposures and potential confounders | Pages 15-18, Table 1&2, Figure 2 |
|  |  | (b) Indicate number of participants with missing data for each variable of interest | N/A |
| Outcome data | 15* | Report numbers of outcome events or summary measures | Ag+: Pages 18-21, Figures 3 -5  Mf: page 24, Table 4  Lymphedema - page 27 |
| Main results | 16 | (*a*) Give unadjusted estimates and, if applicable, confounder-adjusted estimates and their precision (eg, 95% confidence interval). Make clear which confounders were adjusted for and why they were included | Ag+: Pages 18-21, Figures 3 -5  Mf: page 24, Table 4  Lymphedema - page 27 |
|  |  | (*b*) Report category boundaries when continuous variables were categorized | N/A |
|  |  | (*c*) If relevant, consider translating estimates of relative risk into absolute risk for a meaningful time period | N/A |
| Other analyses | 17 | Report other analyses done—eg analyses of subgroups and interactions, and sensitivity analyses | Page 26, Fig 7 |
| Discussion | | | |
| Key results | 18 | Summarise key results with reference to study objectives | Page 28 |
| Limitations | 19 | Discuss limitations of the study, taking into account sources of potential bias or imprecision. Discuss both direction and magnitude of any potential bias | Page 29 & 30 |
| Interpretation | 20 | Give a cautious overall interpretation of results considering objectives, limitations, multiplicity of analyses, results from similar studies, and other relevant evidence | Pages 29-31 |
| Generalisability | 21 | Discuss the generalisability (external validity) of the study results | Page 31 |
| Other information | | | |
| Funding | 22 | Give the source of funding and the role of the funders for the present study and, if applicable, for the original study on which the present article is based | Page 33 |
