# Supplementary material for: Lymphatic filariasis epidemiology in Samoa in 2018: Geographic clustering and higher antigen prevalence in older age groups
Source: PLoS Negl Trop Dis. 2020 Dec 21;14(12):e0008927. doi: 10.1371/journal.pntd.0008927 (PMC7785238; doi:10.1371/journal.pntd.0008927)
Supplement: Attachment — Submitted filename: Lau et al.docx [file pntd.0008927.s008.docx]

This manuscript by Lau et al is very clearly written and provides an excellent epidemiologic framework for first profiling filariasis endemicity and then quantifying the results of MDA treatment – in this case for Samoa, triple-drug (IDA) MDA. By analyzing disease-specific variables (Ag and mf levels for this report) in relation to individuals’ age, sex, household and geo-location, conclusions can be drawn about how best to describe LF-endemic populations so that changes resulting from interventions can be most effectively assessed. The authors’ statistical approaches are particularly robust, both in keeping with their previous publications and in providing a blueprint for others’ studies.

The manuscript has two conceptual drawbacks. First, it would have been Ideal if all the key variables that the authors wanted to assess (including antibody responses in the population and xenomonitoring in vectors) could have been included in the same manuscript or in accompanying manuscripts, but that was not possible. Indeed, even with just Ag and mf determinations, the results are important and the analysis framework provided is an excellent contribution to the literature.

The second drawback for the study is the fact that the key baseline variables assessed (Ag and mf) were evaluated 7-11 weeks *after* the IDA was given. There is no question but that this treatment affected both the Ag and mf levels measured, and certainly the authors must have been very disappointed that the MDA was conducted before the baseline could be established. While the value of their study is still great, this deficiency should be acknowledged again in the first sentence of the Discussion which should be extended to include the following: “…. and 7-11 weeks after MDA was conducted with IDA.” Further, the implications of this pre-baseline treatment should be defined better. The authors make it clear that the mf levels no longer reflect their probable pre-treatment values, but it might be possible to provide a bit more description of how those 7-11 weeks post-MDA affected the antigen levels. The reference cited is not sufficient to support their contention that there was minimal effect on antigen clearance after IDA, but possibly in the analyses of post-IDA changes elsewhere there will be some information on the changes. It is likely that those most lightly infected will lose their positivity first, even in the first 2-3 months post treatment. If the authors cannot come up with a better understanding of the effect of the treatment on Ag levels, they should elaborate on the possibilities a bit more.

Of lesser importance,

- some of the references are incomplete (#s 8, 22, 24, 25, 31, and the ‘personal communication’ cited on line 560).
- There is no need for Figure 8
- The presentation of the lymphedema finding is weak and not really helpful to the manuscript (a simple comment that concern for any LF disease related to the patients was referred to the local medical care would be sufficient).
