# Supplementary material for: Lymphatic filariasis epidemiology in Samoa in 2018: Geographic clustering and higher antigen prevalence in older age groups
Source: PLoS Negl Trop Dis. 2020 Dec 21;14(12):e0008927. doi: 10.1371/journal.pntd.0008927 (PMC7785238; doi:10.1371/journal.pntd.0008927)
Supplement: Attachment — Submitted filename: Lau_SaMELFS 2018 baseline_Response to reviewers_R1_v3.docx [file pntd.0008927.s009.docx]

**Response to reviewers**

**(Reviewers’ comments in black, our responses in blue)**

**Methods**

Reviewer #1: The sampling approaches were mainly appropriate and well executed. 

Line 105: Ivermectin is used for treating onchocerciasis. The triple regimen is indicated for areas where onchocerciasis is not endemic. Can the author add a brief comment why ivermectin was included in the triple regimen and then indicated for areas not endemic for onchocerciasis?

Please see our comment in Lines 100+:

*“In November 2017, WHO endorsed the use of two annual rounds of triple-drug MDA (ivermectin, diethylcarbamazine, albendazole: IDA), which has been shown to be more effective for achieving sustained clearance of microfilariae compared to two-drug regimes [9]. The new triple-drug MDA is recommended for countries where onchocerciasis is not endemic and in areas that i) have not yet started MDA, or ii) have provided fewer than four effective rounds of MDA, or iii) where MDA results have been suboptimal [9].”* The WHO paper can be found here: <https://www.who.int/lymphatic_filariasis/resources/9789241550161/en/>

In Samoa, IDA was used in 2018 because MDA results have been suboptimal.

Line 112: “achievement of GPELF”

Corrected

Line 195: a reference or a rationale is needed for the choice of the sample size determination. Ideally, >80% power is used to determine sample size.

Sample size calculations were based on the WHO document on Monitoring and Epidemiological Assessment of MDA, which recommended use of 75% power for sample size calculations - see Section 7.3.7:

<https://apps.who.int/iris/bitstream/handle/10665/44580/9789241501484_eng.pdf;jsessionid=FA72D7CA65F8DAA6D813523FAC21BED1?sequence=1>

We have added this reference to the paper.

Instead of convenience sampling for logistic reason, it would have been ideal to reduce the n to sample more household in random fashion. However, the other sampling approaches were good.

Our study aimed to compare Ag prevalence between two different age groups: age 5-9 years and ≥10 years. We therefore required roughly equal numbers for each age group. If household surveys alone was used, we would have had to visit two or three times as many households to recruit sufficient 5-9 year-old children. We compared results for 5-9 year-olds sampled in households and convenience surveys, and there were no significant differences. Therefore, we believe that our sampling strategy managed to recruit a representative sample of 5-9 year-olds while optimising operational efficiency.

Hydrocele index is a good indicator. This was not sampled for logistic reasons. However, in private interviews, a simple question posed with option not to respond will be useful in the light of determining the burden of disease.

Most of our field team members were not clinically trained, so we did not consider it appropriate for them to asked detailed clinical questions. Men are often very reluctant to admit to having a hydrocele and interviews were generally conducted in common living areas in households, where there was very little privacy. The prevalence of hydrocoele is significantly lower than antigen prevalence, and our sample size would not have been sufficient to provide an accurate estimate of hydrocoele prevalence anyway. We have amended the Methods to read: *“Due to for logistic* ***and privacy*** *reasons, a hydrocele survey was not conducted.”*

Line 283: If the TAS was performed post MDA, then the Antibody study will be an important component of this publication. Also as indicated by the authors, a post MDA study on Mf is not useful as a baseline data.

We agree with the reviewer that antibodies will provide very useful information. However, antibody prevalence was not a primary objective of this study, and we are still awaiting laboratory work to be conducted. We will share the results of antibody prevalence in future publications once the laboratory work has been completed. We agree that the Mf data are not useful as a baseline, and we have discussed this as a limitation. We have stated clearly in the Discussion:

*“Mf prevalence before MDA was likely to have been higher before MDA, but it was not possible for our study to provide an accurate estimate.”*

Reviewer #2: see attached

Reviewer #3: The objectives were well defined and easy to understand. The study methodology is well detailed, in a structured way and meets the proposed objectives. The criteria for choosing the participants, the selection of the households and children's survey also seems correct.
It presents a robust statistical analysis, according to the type of study.
Ethical issues were granted

Thank you for the positive comments.

**Results**

Reviewer #1: The baseline study readily identified hotspots, but only based on (by purposive sampling) previous knowledge of ongoing transmission in these locations. The rationale for purposive sampling is unclear as this may have skewed the overall reported prevalence. However, a separate analysis was done for randomly selected units, a more useful indices that the purposive sampling and unfortunately the overall prevalence. While this is an interesting finding, reidentifying a hotspot in an already known high transmission location is only academic. The data from random PSUs arrived at the same conclusion that Ag prevalence was higher in the >10yo. This too is an expected outcome since the higher age groups are more likely to be involved in outdoor activities that lead to exposure to infected mosquitoes. And for a chronic disease like LF, time is always a factor.

Thank you for raising this important point. Adjusted Ag prevalence in all ages ≥5 years in randomly selected PSUs ranged from 0% to 10.3%, i.e. we did identify additional hotspot villages through random sampling. The purposive PSUs were identified by the Ministry of Health as areas of previous concern, based on local knowledge about villages with high prevalence, locations of positive children in TAS, etc. All the previous surveys in the past 11 years were school-based TAS in young children, and the data collected were minimal compared to what we collected in the current study. The purposive villages were therefore included here to improve understanding of epidemiology of LF in hotspots, and also to verify the accuracy of local knowledge in identifying hotspots. However, most analysis in the paper (for example by region) used the randomly selected PSUs and we specifically reported results separately for random and purposive PSUs – prevalence estimates from random PSUs to provide an estimate of true prevalence, and prevalence estimates from purposive PSUs to verify the accuracy of local knowledge.

We agree that it was not unexpected for Ag prevalence to be higher in older persons. Currently, the target age group for surveillance is Grade 1-2 children (age approx. 5-9). Our study sought to quantify the difference between two age groups (5-9 and 10+) and provide an evidence base for improving future surveillance strategies. For example, we generated new information on the difference in ratio of prevalence by age between the random and purposive PSU.

There is need to add a comment on the findings of other evaluation studies that may have identified why 10 rounds of MDA failed in Samoa while fewer rounds eliminated LF in some locations. While the authors added their own speculations on why MDA failed over time in Samoa, these were not from scientific studies. If this information is a product of interviews and some form of organized data collection approach, it needs to be stated. Also, previous TAS found that while only NWU failed the first TAS in 2013, all the EUs failed TAS in 2017. This is striking and needs to be studied or commented on to put the current MDA/study in perspective. Such study is important both in planning and evaluating the current MDA.

We agree that it’s important to understand the reasons for ongoing transmission in Samoa despite 10 rounds of MDA. However, no studies have specifically investigated this issue, and the reasons remain unclear. We have therefore discussed potential reasons based on biological plausibility.

We have elaborated on this point in the discussion:

*“The reasons for LF persistence and resurgence in Samoa are unclear. As detailed in the introduction, previous surveys indicated that multiple rounds of MDA did not reduce prevalence thresholds to below recommended levels. Although there have been many surveys in Samoa over the years, none have specifically addressed reasons for ongoing transmission despite completing the recommended rounds of MDA.”*

There is a large amount of data from previous surveys in Samoa, currently being collated and prepared for publication as a paper on the history of LF elimination in Samoa. As mentioned in our Introduction:

*“While LF Ag prevalence (by the rapid Alere ICT) in Samoa was greatly reduced from 4.4% in 1998 to 1.1% in 2004, the WHO-recommended threshold for stopping MDA was not reached [18,19] and further nationwide MDAs were distributed in 2008 and 2011 [20,21]” and “In 2013, TAS in school children aged 6-7 years identified that transmission was still occurring in NWU [22]. Two more rounds of two-drug MDA were distributed in NWU in 2015 and 2017 [23,24]. A repeat TAS in 2017 showed that all three EUs failed to meet elimination targets and further nationwide MDA was required [24].”*

Apart from these programmatic surveys, no other population representative LF human surveys had been done until our current study, and none of the previous surveys addressed reasons for ongoing transmission.

The authors showed confidence intervals without p-values in all the important data. Although this study is more descriptive, the p-values will orient the reader to the significance of the identified differences and associations.

While it is true that p values are conventionally used to assess statistical significance, there is now a greater emphasis on reporting the effect size or group differences with test statistics and/or confidence intervals. As p value is very dependent on the sample size, differences can be statistically significant based on p value while being not epidemiologically or clinically important. If 95% confidence intervals of two estimates do not overlap, then the estimates are statistically significantly different.

For prevalence estimates where there are multiple subgroups (e.g. Figures 3 &4), we believe that it is more useful to present the point estimate and 95% CI so that differences between any pairs of results can be easily compared. While it is possible to report p values for every pairwise comparison, this is cumbersome and does not add much to reporting 95% CI. We have included p values for a few more comparisons where we believe it adds to the interpretation of results.

Line 337: Please check the sentence “…... while a there..”

Corrected

Line 553: change ‘it’s’ to ‘it is’ or ‘it was’

Amended

For all figures, please provide a caption. In addition, move the figure labels below the figure.

Amended

Reviewer #2: see attached

Reviewer #3: The results are clear and well supported by the tables and figures. The most relevant data were clearly presented. The analysis presented corresponds to the defined plan.

**Conclusions**

Reviewer #1: Authors found that sampling 5-9 age group was not as useful as sampling >10 ager group in determining prevalence and cut off for stopping MDA. This appear to not be concomitant with the 6-7 ager group recommended by GPELF. More comment will be interesting on how the 6-7 age group has performed in other areas/studies, maybe a few comments on the previous study cited by the authors.

The 5-9 year-old age group was used for a number of reasons:

- The GPELF M&E guidelines recommend school-based surveys of Grade 1 and 2 children, who are mostly 6-7 years old, but in some countries, this may include children in the age range of 5-9 years.
- For onchocerciasis, the 5-9 year-old age group is used for surveillance^[1]^, and there are practical advantages in aligning age groups used for M&E of different diseases. Although there is no onchocerciasis in the Pacific Islands, the findings of our studies have implications for LF programmes globally.
- Another aim of the study (not reported here) was to determine whether antibody prevalence could be useful indicators of areas of ongoing transmission, especially in young children. We therefore wanted to capture a wider age range in young children to investigate the best serological indicators to use, as well as the best age group to target for school-based surveys.

Reviewer #2: see attached

Reviewer #3: The conclusions reflect the results obtained, which at the same time were well discussed, according to the information described in the literature.
They presented the limitations of the study, which helps to understand some of the results.
This is a topic of high importance from public health point of view, as mentioned throughout the article. This work clearly contributes to the increase in knowledge of the epidemiological situation of lymphatic filariasis in Samoa, thus allowing new actions by governmental entities to control the disease.

Thank you for the positive comments.

**Editorial and Data Presentation**

Reviewer #1: For all figures, please provide a caption. In addition, move the figure labels below the figure.

Amended

Reviewer #2: see attached

Reviewer #3: There are some aspects that should be clarified, upgrading the work. In the introduction, it is mentioned that lymphatic filariasis is “caused by three species of filarial worms (Wuchereria bancrofti, Brugia malayi and B. timori). However, it was not mentioned whether the 3 species existed in Samoa. In the results, is refered that the number of microfilaria was counted, but it is not mentioned the species of microfilaria, which from an epidemiological point of view, would be important. Is that data available?

We have added the following information in the Introduction:

*“Wuchereria bancrofti* is the only species of filarial worm that causes LF in Samoa and infection is transmitted by multiple species of *Aedes* mosquitoes, predominantly *Aedes polynesiensis*.”

I think that the last paragraph of the results, related to lymphedema, is not relevant for this paper, as well as the figure 8. Despite of the clinical relevance, this is not included in the estudy.

Assessment of lymphedema was the third aim of our study (line 131):

*“…….iii) assess the prevalence of limb lymphedema in those aged ≥15 years.”*

In the references: Numbers  10, 20, 23, 28, 30, the “accession date” is missing; in numbers 22 and 24, the “accession date” and the web link are missing.

The references have been corrected.

**Summary and General Comments**

Reviewer #1: Lau et al here reports “Lymphatic Filariasis Epidemiology in Samoa in 2018: Geographic Clustering and Higher Antigen Prevalence in Older Age Groups”, a baseline study prior to an MDA program with three ivermectin, DEC and albendazole. This is an important study that serves as both evaluation of previous intervention efforts which the authors allude started in the 1960s, it also serves as a reference point for the evaluation of the ongoing MDA. The report is also well written and well organized. The sampling approaches were mainly appropriate and well executed. However, a few observations on how this study can be improved have been included. While some of these comments are probably not within the scope of what can be modified at this point, efforts should be made to at least address these as limitations of the study within the paper.

Thank you for the positive comments. We agree that some of the points raised were outside of the scope of this study. We have addressed each of the comments above.

Reviewer #2: see attached

Reviewer #3: I think this is a work of the greatest relevance from an academic and scientific point of view. The theme is actual, and about a debilitating disease, with a high impact on society. The article is well written, well structured and easy to understand. It presents a good introduction, well-defined objectives, appropriate methodology to the proposed objectives. The results are relevant, supported by a robust statistical analysis. A good discussion, with well-founded conclusions, supported by a good and actual Bibliographic references list

Thank you again for the supportive comments.

**Reviewer 2 attachement**

This manuscript by Lau et al is very clearly written and provides an excellent epidemiologic framework for first profiling filariasis endemicity and then quantifying the results of MDA treatment – in this case for Samoa, triple-drug (IDA) MDA. By analyzing disease-specific variables (Ag and mf levels for this report) in relation to individuals’ age, sex, household and geo-location, conclusions can be drawn about how best to describe LF-endemic populations so that changes resulting from interventions can be most effectively assessed. The authors’ statistical approaches are particularly robust, both in keeping with their previous publications and in providing a blueprint for others’ studies.

The positive comments are much appreciated.

The manuscript has two conceptual drawbacks. First, it would have been Ideal if all the key variables that the authors wanted to assess (including antibody responses in the population and xenomonitoring in vectors) could have been included in the same manuscript or in accompanying manuscripts, but that was not possible. Indeed, even with just Ag and mf determinations, the results are important and the analysis framework provided is an excellent contribution to the literature.

We agree that antibody and xenomonitoring results are very important, and would add greatly to the results presented here. Antibody prevalence was not a primary objective of this study, and we are still awaiting laboratory work to be conducted. We will share the results of antibody prevalence in future publications once the laboratory work has been completed. We decided to publish the xenomonitoring results in a separate paper because they would be too complex to include here, and detract from the Ag and Mf results. Our xenomonitoring results include extensive data for different mosquito species by PSU and region, as well as comparison of mosquito infection prevalence before and after the first round of IDA. We hope to complete this publication soon.

The second drawback for the study is the fact that the key baseline variables assessed (Ag and mf) were evaluated 7-11 weeks *after* the IDA was given. There is no question but that this treatment affected both the Ag and mf levels measured, and certainly the authors must have been very disappointed that the MDA was conducted before the baseline could be established. While the value of their study is still great, this deficiency should be acknowledged again in the first sentence of the Discussion which should be extended to include the following: “…. and 7-11 weeks after MDA was conducted with IDA.”

We agree that the timing of the survey was not ideal, and it was certainly disappointing that we could not conduct the baseline before the start of IDA. We have acknowledged this under limitations, and as suggested by the reviewer, we have included additional information about the timing of the survey in the first paragraph of the discussion.

Further, the implications of this pre-baseline treatment should be defined better. The authors make it clear that the mf levels no longer reflect their probable pre-treatment values, but it might be possible to provide a bit more description of how those 7-11 weeks post-MDA affected the antigen levels. The reference cited is not sufficient to support their contention that there was minimal effect on antigen clearance after IDA, but possibly in the analyses of post-IDA changes elsewhere there will be some information on the changes. It is likely that those most lightly infected will lose their positivity first, even in the first 2-3 months post treatment. If the authors cannot come up with a better understanding of the effect of the treatment on Ag levels, they should elaborate on the possibilities a bit more.

We agree with the reviewer that it would be very useful to be able to estimate true prevalence of Ag and Mf at baseline. We have reviewed the published evidence on Mf and Ag clearance post-IDA, and some unpublished results from PNG. Unfortunately, there isn’t sufficient evidence for us to accurately estimate baseline prevalence based on our results at 7-11 weeks post-IDA. Most studies that used IDA measured Ag after 12 months or longer, and studies that measured Ag prevalence after 1-3 months did not use IDA.

We have made it clearer in the first paragraph of the Discussion that your results were from a survey conducted 7-11 weeks after the first round of triple-drug MDA.

We have also added the following paragraph to the Discussion:

*“We expect the timing of the survey to have affected Mf prevalence and to a lesser degree Ag prevalence. Mf clearance can occur very rapidly after treatment; Thomsen et al reported Mf clearance in 91.7% of participants (n=12) one week after IDA [37], and McCarthy et al reported that 95.8% of participants (n=24) were Mf-negative one week after taking DEC alone [38]. Previous studies have reported varying rates of Ag clearance after treatment with IDA. A study in Cote d’Ivoire found that after a single dose of IDA, mean circulating filarial Ag (measured by ELISA) reduced by 70% at six months and 75% at 12 months, while FTS score reduced by 50% at 6 months and 55% at 12 months [39]. Similar results were observed in PNG, where a 50% reduction in Og4C3 Ag level was observed at 12 months [37]. Studies in PNG [40,41] and Haiti [42] reported that after a single dose of IDA, 95%-97% and 79.5% of Ag-positive participants were still positive after 12 months, respectively. These findings suggest that after treatment with IDA, Ag-positivity is likely to persist for months in the majority of people. None of the studies that used a single dose of two-drug or three-drug combinations investigated Ag clearance rates around the 7-11 week period post-treatment (when our survey in Samoa was conducted), so there is currently insufficient knowledge about Ag clearance in the immediate post-MDA period for us to accurately estimate true prevalence at baseline. However, we can be confident that the baseline Ag and Mf prevalence before the first round of triple-drug MDA in Samoa would have been at least as high as our reported values.”*

Of lesser importance,

- some of the references are incomplete (#s 8, 22, 24, 25, 31, and the ‘personal communication’ cited on line 560).

The references have been corrected, and we have removed the reference to ‘personal communication’.

- There is no need for Figure 8

Figure 8 has been removed

- The presentation of the lymphedema finding is weak and not really helpful to the manuscript (a simple comment that concern for any LF disease related to the patients was referred to the local medical care would be sufficient).

Even though we did not identify an LF-related lymphedema, the results have been included here because assessment of lymphedema was one of the aims of our study (line 131). We have abbreviated this section and removed details of the cases with lymphedema from other causes.

1. Report of the 1^st^ Meeting of the WHO Onchocerciasis Technical Advisory Subgroup. Geneva, Switzerland. Oct 2017. <https://www.who.int/onchocerciasis/resources/WHO-CDS-NTD-2018.05/en/>
